# Impact of biofeedback therapy for pelvic floor-related constipation to improve sexual function 

**Published:** 2020

**Authors:** Seyedshahab Banihashem, Nasrin Chalakinia, Pegah Eslami, Mehran Mahdavi Roshan, Ali Kheradmand, Saeed Abdi, Somayeh Motazedian, Maryam Nasserinejad, Mohammad Reza Zali

**Affiliations:** 1 *Department of Psychosomatic Medicine, Taleghani Hospital Research Development Committee, Shahid Beheshti University of Medical Sciences, Tehran, Iran*; 2 *Psychiatry and Behavioral Research Center, Mashhad University of Medical Sciences, Mashhad, Iran*; 3 *Gastroenterology and Liver Diseases Research Center, Research Institute for Gastroenterology and Liver Diseases, Shahid Beheshti University of Medical Sciences, Tehran, Iran *

**Keywords:** Sexual dysfunction, Biofeedback, Pelvic floor disorder, Constipation

## Abstract

**Aim:**

The purpose of this study was to investigate the effect of biofeedback therapy on constipation to improve sexual function among the female population with pelvic floor hypertonicity.

**Background:**

It appears that pelvic floor disorder could lead to sexual complaints. Unfortunately, there are few data on the correlation between pelvic floor-related constipation and sexual disorders. The biofeedback role as a conservative method in improving the health status in these patients is conflicting.

**Methods:**

Forty-two eligible women were included in the study. The exclusion criteria were not being sexually active, not having functional constipation according to Rome IV criteria, and having other psychiatric issues, according to DSM4TR criteria. All participants were treated using biofeedback in eight sessions, during two months. Before and after the treatment, they were analyzed by pelvic floor impact questionnaire, pelvic floor Distress Inventory, and Short Scale Personal Experiences Questionnaire (SPE Q).

**Results:**

Biofeedback significantly improved orgasm, arousal, and dyspareunia (respectively P = 0.001, P = 0.001, P = 0.001). However, there was no significant improvement in libido and partner satisfaction domains (respectively P = 0.132, P = 0.341). Significant negative correlations were detected between the age and sexual function. On the other hand, there was no negative relationship between vaginal delivery as well as cesarean delivery and different components of sexual function.

**Conclusion:**

It seems the improvement in pelvic floor muscle hypertonicity leads to sexual satisfaction. Nevertheless, more data are required to prove this correlation.

## Introduction

 Sexual dysfunction is a common complaint in females above 40 years of age (1). Sixty-five percent of women have reported at least one related complaint in their lifetime (2). Sexual health as a part of the quality of life is defined as physical, emotional, mental, and social well-being, concerning sexual activity (3). The female sexual function includes sexual interest, arousal, orgasm, dyspareunia, and complete intercourse (4). Nearly 25% of females are not interested in sex, 20% do not experience orgasm, and 10% have dyspareunia worldwide (4).

Recently, pelvic floor dysfunction has been regarded as one of the most important risk factors of sexual dysfunction in the elderly population (5, 6). The incidence rate of this anatomical disorder is about 33% worldwide (2). Pelvic floor dysfunction encompasses pelvic organ prolapse, chronic pain, and other abnormalities in urination, defecation, and sexual function (7). 

Constipation related to pelvic floor dysfunction is known as obstructive constipation. Pelvic floor dysfunction accounts for 7% of functional constipation (8). Although several studies have assessed the correlation between different components of pelvic floor dysfunction and sexual dysfunction, limited and conflicting data exist on the role of obstructive constipation in sexual dysfunction (9). In addition, no established treatment is available for obstructed constipation. Previous studies have shown that biofeedback therapy is more effective in improving obstructive constipation than comparison to clinical and surgical therapies (2). Murad- Regadas et al. (2) reported that biofeedback therapy along with clinical treatment significantly improved obstructive constipation. 

This study explores the dearth of research on the impact of biofeedback therapy on obstructed constipation to improve sexual function across the female population. 

## Methods

**Study design**

Between December 2017 and November 2018, among the women who attended Taleghani gastrointestinal clinic, the patients with constipation diagnosis according to the Rome IV questionnaire and sexual disorders at the same time were evaluated in terms of pelvic floor dysfunction by manometry (10). Among them, patients with pelvic floor muscle hypertonicity were included in this trial, with no age limitation. The exclusion criteria were as follows: 1. The women who were not sexually active. 2. Women who did not have functional constipation according to Rome IV criteria. 3. Women who had other psychiatric issues, according to DSM4TR criteria. 

In the first step, all patients were evaluated by a standardized history and clinical examination. The history included questions about the age, marital status, history of constipation according to Rome IV criteria, other signs and symptoms related to pelvic floor dysfunction, number and modality of deliveries, as well as previous surgical operations. The severity of pelvic floor dysfunction was determined according to the completed validated short form of Pelvic Floor Distress Inventory (PFDI – 20)(11) and Pelvic Floor Impact Questionnaire (PFIQ – 7) (11). Then, all patients were evaluated according to DSM4TR, where the patients with psychiatric dysfunctions were excluded. 

Next, the sexual function of all participants was assessed based on the sexual personal experience questionnaire, a validated psychometric tool developed to measure sexual function in women for population-based and clinical trial research. According to the Short Scale Personal Experiences Questionnaire(SPEQ)(12), four aspects of sexual function were evaluated, including libido (desire), sexual arousal, orgasm, dyspareunia, sexual partner satisfaction (13). Then, the patients with sexual disorders were evaluated in terms of pelvic floor muscle hypertonicity via manometry. Finally, the biofeedback therapy was done in the same situation and with the same physicians for all participants. Before the treatment, all participants were given complete information about the procedure. The participants were treated once a week up to eight sessions.

**Assessment of constipation and obstructed constipation**

Obstructive constipation in this study was defined as functional constipation (according to Rome IV criteria) plus positive results from manometry. Manometry recorded sphincter function, recto-anal reflex activity, and rectal pressure during the defecation plus the hypertonicity of the pelvic floor muscles (14). 

**Treatment**

A total of 42 eligible participants received eight-session biofeedback therapy during two months. Each session of biofeedback therapy included practicing expulsion by inserting an 8-lumen catheter followed by squirting and relaxing periods. After two months of biofeedback therapy, the participants were evaluated by Pelvic Floor Impact Questionnaire 7 (PFIQ7), the Pelvic Floor Distress Inventory (PFDI-20), and SPE Q. with the new results compared with the previous ones. The measures of improvement were also reported.

**Statistical analysis**

The total number of patients needed to show a 30% success rate differs from the previously established success rate of approximately 50% was determined in order to detect a clinically significant difference. The minimum participants for this study were estimated about 42. 

The analysis included descriptive statistics (means, standard deviation, minimum and maximum values, and percentages). Correlations were measured by Pearson’s correlation coefficient (r) and Spearman’s Rho (p). Also, to determine confounding factors in the study, a bivariate analysis was performed. *P *< 0.05 was considered statistically significant. All analyses were done using SPSS (SPSS Inc. Released 2006. SPSS for Windows, Version 15.0. Chicago, IL: SPSS Inc.)

**Ethical approval**

All included patients were informed about the study. Furthermore, the participants were allowed to exit from the study at any of its steps. The study was approved by the ethics committee of Shahid Beheshti University of medical sciences (It.sbmu.retech.rec.1398.619). 

## Results

Forty-two women were included in this study. Baseline demographic and clinical characteristics are shown in Tables 1-3. 

**Table 1:** Characteristics of the women with comorbid obstructive constipation and sexual disorder

variables	mean	median	Std^†^	min	max
Age (year)	44.09	41.50	±13.09	19.00	71.00
BMI* (kg/m²)	26.01	25.65	±3.40	19.72	32.86

*Body Mass Index, ^†^Standard Deviation

**Table 2:** Number and frequency of vaginal delivery of comorbid obstructive constipation and sexual disorder

Number of deliveries	Frequency	Percentage
0	21	50
1	1	2.4
2	7	16.7
3	7	16.7
4	5	11.9
8	1	2.4
Total	42	100

**Table 3:** Menopausal situation of comorbid obstructive constipation and sexual disorder

	Frequency	Percentage
Positive	15	35.7
Negative	27	64.3
Total	42	100

The mean age of the participants was 44.09±13.09 years (ranging from 19 to 71 years). Menopausal women comprised 35.7% of the study population. The mean Body Mass Index (BMI) was 26.02±3.41 (range: 19.72-32.86). Half of the participants had at least vaginal delivery once, and only 9.5% of the participants had a positive history of surgery. The efficacy of biofeedback therapy was evaluated in different components of sexual function, including arousal, libido, orgasm, dyspareunia, and sexual-partner satisfaction. No significant improvement in libido was found after two months of biofeedback therapy (P=0.132). However, the Intervention led to significant improvements in arousal, orgasm, and dyspareunia domains (P=0.001). Sexual partner satisfaction, according to all of the three questionnaires did not increase significantly after the intervention (first questionnaire (P=0.285), second questionnaire (P=0.102), third questionnaire (P=0.655). Overall, biofeedback therapy did not significantly improve the sexual partner satisfaction (p = 0.340) (Table 4).

**Table 4:** Analytical statistical results before and after biofeedback therapy in women with comorbid obstructed constipation and sexual disorder

Index	biofeedback therapy		p-value*
	Before	After	
Libido/desire	1.59±0.70^†^	1.71±0.63	0.132
Arousal	5.95±2.80	7.66±2.67	0.001
Orgasm	3.14±1.89	3.54±1.82	0.001
Dyspareunia	3.38±1.63	2.21±1.15	0.001
Sexual partner satisfaction	11.04±2.48	11.28±2.23	0.340

*P-value less than 0.05 was considered to be statistically significant; ^† ^mean ± standard deviation

There were significant negative correlations between the age of the participants and all compartments of sexual function except dyspareunia (r(libido)=0.022, arousal < 0.001, orgasm < 0.001, dyspareunia= 0.507). Also, there were significant inverse correlations between BMI and two components of sexual function, including orgasm and arousal (respectively 0.005, 0.001). No significant inverse correlation was found between the vaginal delivery and different compartments of sexual function. In addition, a similar result was found between caesarean delivery and sexual function. Furthermore, the positive history of surgery did not affect the sexual function.

## Discussion

Sexual health is an important and inseparable part of the quality of life (15). It has different components including orgasm, libido, dyspareunia, arousal, and sexual partner satisfaction (16). About 67% of women with sexual complaints seek help from gynecologists at least once in their lifetime (7). Several previous studies have shown a relationship between pelvic floor dysfunction and sexual disorders (17). However, the pathophysiology of this relationship is still unknown (1). Pelvic floor dysfunction includes heaviness or pressure in the pelvic floor area, vaginal bulging, pelvic pain, urinary or fecal incontinence, and obstructive constipation (9). Ryan J. Li-Yun-Fong et al. (18) in their retrospective study reported that of 755 participants with pelvic floor dysfunction, 70% had obstructive defecation. This study and other similar studies have indicated that obstructive constipation comprises a major part of pelvic floor dysfunction. The possible cause of obstructive constipation appears to be pelvic floor muscle hypertonicity which leads to sexual disorders (7). 

Overall, there are two types of therapy for pelvic floor disorders, including surgical and conservative therapies (5). Studies have shown that although the surgical treatment improves the main anatomical dysfunction, it increases the risk of nerve damage, which, in turn, leads to sexual disorders. Recently, biofeedback has been noticed as a conservative therapy in pelvic floor dysfunction (19). Studies have reported that approximately more than half of patients with pelvic floor dysfunction completely respond to biofeedback (10). Despite the high prevalence of pelvic floor dysfunction, data are limited regarding the effects of the treatment of obstructive constipation on sexual activity. Pellino et al. (20) in their study reported that fecal incontinence and constipation had significant negative effects on sexual function. In another study, Bortolami et al. (7) worked on the correlation between pelvic floor dysfunction and sexual disorders. They concluded that sexual disorder significantly correlated with the pelvic floor muscle hypertonicity and age in patients undergoing physical therapy as well as rehabilitation; thus, the hypertonicity effects were higher in reproductive age women. 

The current study evaluated the effect of biofeedback in women with obstructive constipation on the improvement of sexual function. Our results showed a direct correlation between the severity of pelvic floor dysfunction and sexual disorders. The results also revealed that biofeedback therapy led to improved sexual function among women with obstructive constipation in three domains of sexual function, including arousal, dyspareunia, and orgasm. However, the treatment was observed to have no significant effect on sexual satisfaction and libido. Further, the treatment demonstrated a more significant efficacy among menopausal women compared to the fertile group. Previous studies reported age, BMI, and types of delivery as discrete random variables affecting sexual function. The mean age of the participants was 45 years old, and the results showed a significant inverse correlation between the age of the participants and all domains of sexual function, except dyspareunia. It seems age played an important role in pelvic floor dysfunction and thus led to sexual disorders. The continuous follow-up with regard to pelvic floor dysfunction and sexual disorders in elderly and menopausal women helped us detect more related cases and improve the quality of the sexual life of the population. Note that the participants were, on average, overweight with the mean BMI of 26.02±3.4. In this study, a significant inverse correlation was detected between BMI with orgasm and arousal. In a case-control study carried out by Dilek Bilgic et al. (6) it was shown that obese women had longer pelvic floor dysfunction symptoms and their quality of life was inferior compared to non-obese women. Kalaivani Ramalingam et al. (21) in a review article evaluated the correlation between obesity and different components of pelvic floor disorders. With regard to the relationship between constipation and obesity, they believed that decreasing BMI could help improve the symptoms of constipation in patients with constipation associated with pelvic floor dysfunction. They also explained the relationship between obesity and sexual disorders according to previous related research. They stated that weight loss could improve sexual function in all stages including arousal, lubrication, libido, and sexual satisfaction. Contrary the previous studies, our study showed no association between the delivery types and different domains of sexual function. Ian Milsom et al. (3) explained childbirth delivery as an important risk factor for pelvic floor dysfunction. Skinner EM et al. (8) in their literature review regarded the sexual disorder as a side event of the vaginal delivery. Possibly, the small population of our study led to conflicting results. More studies are recommended accordingly. 

The present study had several limitations. First, the relatively small sample size of our study may have reduced the power to detect statistically significant differences between various factors. Secondly, owing to the single-center experience of this study, it may not be generalized to other centers. Furthermore, the lack of the control group could be considered as another important limitation of this study. Therefore, the results of the present study should be interpreted in the context of its limitations. 

According to the current study, it seems biofeedback therapy in obstructive constipation could lead to improved sexual function, including orgasm, dyspareunia, and arousal. Thus, psychologists and gynecologists are recommended to consider pelvic floor muscle hypertonicity as an important cause of sexual disorders among female patients. Nevertheless, more investigations are required to prove the biofeedback as an effective treatment in pelvic floor dysfunction with sexual complaints.

## Funding 

This research did not receive any specific grants from funding agencies in the public, commercial, or not-for-profit sectors.

## Conflict of interests

The authors declare that they have no conflict of interest.
